# Direct Chloroplast Sequencing: Comparison of Sequencing Platforms and Analysis Tools for Whole Chloroplast Barcoding

**DOI:** 10.1371/journal.pone.0110387

**Published:** 2014-10-17

**Authors:** Marta Brozynska, Agnelo Furtado, Robert James Henry

**Affiliations:** Queensland Alliance for Agriculture and Food Innovation, The University of Queensland, Brisbane, Queensland, Australia; Sabanci University, Turkey

## Abstract

Direct sequencing of total plant DNA using next generation sequencing technologies generates a whole chloroplast genome sequence that has the potential to provide a barcode for use in plant and food identification. Advances in DNA sequencing platforms may make this an attractive approach for routine plant identification. The HiSeq (Illumina) and Ion Torrent (Life Technology) sequencing platforms were used to sequence total DNA from rice to identify polymorphisms in the whole chloroplast genome sequence of a wild rice plant relative to cultivated rice (cv. Nipponbare). Consensus chloroplast sequences were produced by mapping sequence reads to the reference rice chloroplast genome or by *de novo* assembly and mapping of the resulting contigs to the reference sequence. A total of 122 polymorphisms (SNPs and indels) between the wild and cultivated rice chloroplasts were predicted by these different sequencing and analysis methods. Of these, a total of 102 polymorphisms including 90 SNPs were predicted by both platforms. Indels were more variable with different sequencing methods, with almost all discrepancies found in homopolymers. The Ion Torrent platform gave no apparent false SNP but was less reliable for indels. The methods should be suitable for routine barcoding using appropriate combinations of sequencing platform and data analysis.

## Introduction

A universal plant barcoding method based upon sequencing a specific gene or combination of specific genes has proven elusive. Analysis of different plant groups has required the use of different genetic loci. The emergence of next generation sequencing (NGS) technology allowing whole chloroplast genomic DNA sequencing has provided the opportunity to use the whole chloroplast genome sequence as a barcode [1]. The chloroplast has unique features found in all green plants, conserved sufficiently to be readily aligned for comparison of different samples and large enough to contain variation, which allow species or sub species distinction across the seed plants [2]. Analysis of complex mixtures of plants has applications in many ecological studies [3].

A generic method for rapid and cost effective DNA-based identification of plants at this level (species or sub-species) will find wide application in industry and research [4], [5]. Industrial applications will include the identification of plant components in whole and processed foods and the management of food processing to ensure food safety and authenticity of labelling [6]. This will complement the use of next generation sequencing to screen for the presence of pathogenic microbes [7]. Protection of intellectual property rights associated with plant varieties will also be simplified by a standard approach to plant identification. These tools will also support protection of biosecurity and management of wild plant populations of rare or threatened plants.

Early analysis relied on either specific amplification of the chloroplast genome or separation of chloroplasts from nuclear and mitochondrial DNA before chloroplast DNA isolation and sequencing the amplified or cloned product derived from PCR amplification [8]. However, the universal amplification of chloroplast sequences from all species has proven difficult and chloroplast purification is laborious and not perfect [9], [10]. Recently a simple approach involving NGS of a total genomic DNA preparation was proposed [11]. This method relies on successful extraction of chloroplast genome sequence reads from total genomic DNA reads and their assembly to form a consensus sequence for the sample that can be used as a barcode. Reference-guided mapping of sequence reads and *de novo* assembly have been used to generate these whole chloroplast barcodes [12].

DNA sequencing platforms differ in their throughput and accuracy [13]. Accuracy of a DNA sequence assembled using NGS reads is dependent on the read length, sequencing depth, sequence coverage or width and evenness of coverage but also on the accuracy of the sequencing platform and the assembly and analysis pipeline [14]. We compared two different sequencing technologies, the Illumina platform that uses sequencing-by-syntheses (SBS) chemistry and the Ion Torrent that is based on semiconductor sequencing technology. Sequencing on both platforms commences with library preparation, which involves fragmentation of total genomic DNA, purifying to uniform and desired fragment size and ligation to sequencing adapters specific to the platform [15], [16]. In Illumina systems the fragments are subsequently denatured and fixed on the surface of a proprietary glass flowcell, followed by solid-phase amplification (bridge amplification). As a result clusters are created which contain clonally amplified DNA templates. Next the clusters are sequenced in parallel using four fluorescently labelled nucleotides with reversible dye terminators. After every sequencing cycle and base incorporation to the template the polymerization terminates, a CCD (charge-coupled device) captures the fluorescent signal and identifies the base. After the base call the dye is cleaved and the sequencing process continues. This technology was first implemented in an instrument called Genome Analyzer (GA) with subsequent releases of improved series up to GAIIx. The next major system launched by Illumina was the HiSeq2000 with improved output and read length. Data from both GAIIx and HiSeq was used in the current study.

In Ion Torrent systems the prepared libraries are immobilized to beads and amplified using emulsion PCR which takes place within microdroplets of aqueous solution and oil [17]. During the sequencing individual bases are incorporated by the action of a DNA polymerase. As a result of this reaction a proton is released and the resulting change in pH is measured. The reaction occurs in a proprietary chip, which acts as a pH meter. Unlike Illumina, this system does not use fluorescent dyes and light detection, shortening the sequencing time. The Ion Torrent instrument used in the study was a Personal Genome Machine (PGM).

We examined the reliability of these two different sequencing platforms coupled with different data analysis approaches when applied to obtaining the chloroplast consensus sequences of a wild rice genotype and a cultivated rice genotype. Although the Ion Torrent platform is widely used for sequencing and limited published data is available on its application for transcript [18] and amplicon [19] sequencing in plants, it has not previously been applied to plant genomic DNA analysis. The most common applications of the Ion Torrent system have been to small genomes (bacterial samples) and targeted sequencing of amplicons or transcripts, whereas the Illumina has been widely used for applications including whole human genome re-sequencing, *de novo* plant and animal genome sequencing, exome, transcriptome (RNA-Seq) and metagenomics investigations. The chloroplast genome represents a specific sequencing challenge with the presence of an inverted repeat. This study compares total DNA sequencing with the Illumina and Ion Torrent platforms and options for analysis of the sequences generated to produce a whole chloroplast genome sequence as a barcode for plant identification. Whole genome NGS was carried out on two rice genotypes: *Oryza sativa* spp. *japonica* var. Nipponbare referred to here as the reference-rice-genotype (R-rice-genotype) as its chloroplast sequence (reference-sequence) is publicly available and an Australian wild rice genotype sample (W-rice-genotype).

## Materials and Methods

### Plant materials

Seeds of rice (*Oryza sativa* cv. Nipponbare), referred to as reference-rice-genotype (R-rice-genotype), were germinated and entire seedlings were used for DNA extraction. Leaf tissue from a wild rice plant, referred to as wild-rice-genotype (W-rice-genotype) collected from a field containing a population of perennial wild rice (*Oryza rufipogon*-like) at Abattoir Creek in North Queensland, was used for DNA extraction [20].

### DNA extraction

DNA from leaf tissue of the W-rice-genotype was extracted using modification of the CTAB method [21]. DNA from seedlings of the R-rice-genotype was extracted as described for the W-rice-genotype but with further purification as described by [11].

### Sequencing

The Illumina and the Ion Torrent sequencing platforms were used for the shotgun sequencing of total genomic DNA of both rice samples. The wild rice sample was sequenced on the Ion Torrent (200 bp reads) and the Illumina HiSeq platform (100 bp paired end reads). The cultivated rice genotype was subjected to sequencing using the Ion Torrent (200 bp reads) platform while Illumina reads (36 bp paired-end), generated on the GAII, were sourced from available archived data [11]. Sequencing on the Illumina HiSeq 2000 (Illumina, San Diego, CA, USA), to generate 100 bp paired end reads with an average library insert size of 500–600 bp, was outsourced to the Australian Genome Research Facility (AGRF, Melbourne, Australia). For sequencing using the Ion Torrent platform, genomic DNA was sheared using the Covaris S220 instrument (www.covarisinc.com) and used for preparing sequencing libraries according to the standard Ion Torrent PGM protocol. The resulting individual DNA libraries were quality checked and quantified on the Agilent 2100 Bioanalyzer using the High Sensitivity DNA kit (Agilent). Following template amplification and enrichment on the Ion OneTouch2 (Ion OneTouch 200 Template Kit v2; #4478316) and OneTouch2 ES instruments, each sample was loaded onto one PGM #318 chip and sequenced using Ion PGM Sequencing 200 Kit v.2 (#4482006) according to manufacturer’s protocol.

### Data analysis – consensus

Raw reads from both sequencing platforms, PGM Ion Torrent and Illumina, were imported to CLC Genomics Workbench 6.0 (CLC-GW) (www.clcbio.com) and read statistics assessed using sequencing data quality control, followed by read trimming for quality, length and presence of ambiguous bases. Ion Torrent reads were trimmed with the quality score limit set to 0.05 (which corresponded to PHRED quality value >15) and a minimum read length of 30 bp. Reads from both Illumina platforms, GAII and HiSeq, were trimmed with a quality score limit of 0.01 (which corresponded to PHRED >22) and the same minimum read length of 30 bp. Ion Torrent data was trimmed at a slightly lower quality value due to the lower average quality compared to the Illumina reads (Table S1; avg. PHRED for Ion Torrent reads: 25 and 26, and avg. PHRED for Illumina: 29 and 32). The Illumina HiSeq platform generates sequencing data fifty to sixty times more in order of magnitude as compared to the Ion Torrent data. Thus, in order to compare Ion Torrent and Illumina HiSeq platforms at similar coverage, a subset of HiSeq Illumina reads for the wild rice sample and matching the Ion Torrent read numbers for that same sample, was randomly extracted from the whole reads set. Ion Torrent raw reads were also alternatively trimmed using the Torrent Suite Software version 3.6 using default analysis settings with the following modifications: the quality-based trimming of the sequencing reads was adjusted to predicted-PHRED-score 17 over the sliding window of 20 bp (default values 15, and 30 respectively) and the minimal length filter for reported reads was set to 20 (default 8).

Consensus sequence generation using the mapping tool was carried out as follows. The CLC-GW was used to map trimmed reads to the chloroplast genome sequence of *Oryza sativa* spp. *japonica* var. Nipponbare (GenBank accession – GU592207) used as the reference-sequence. The mapping process involved the following parameters: mismatch cost 2, insertion cost 3, deletion cost 3, length fraction 0.9, similarity fraction 0.9, and global alignment setting. Non-specific matches were also mapped randomly to the reference genome. Consensus sequences were extracted from all mapping runs giving five sequences in total; two consensus sequences for *O. sativa* Nipponbare each derived from the Illumina GAII reads and the Ion Torrent reads, and three consensus sequences for the wild rice sample each derived from the Illumina HiSeq sequence reads, Illumina HiSeq sequence subset-reads and the Ion Torrent reads. Conflicts between reads were resolved by voting for the majority of the reads at the given position. Consensus sequence were aligned to the reference sequence and analysed for SNPs and indels.

Consensus sequence generation using the assembly tool was carried out as follows using both the CLC-GW and the Torrent Suite Software. The CLC trimmed reads from both platforms were assembled in CLC-GW using the *de novo* assembly tool with the following parameters: automatic word and bubble sizes with 200 bp for minimum contig length. Moreover the reads were mapped back to contigs (mismatch cost 2, insertion cost 3, deletion cost 3, length fraction 0.9, and similarity fraction 0.9) and contigs were updated based on the results. The trimmed Ion Torrent reads were also assembled in the Torrent Suite Software using the Assembler plugin v.3.4.2 utilizing the MIRA assembly algorithm (http://sourceforge.net/projects/mira-assembler/). The assembly was performed in the reference-assisted mode using the reference-sequence. All contigs generated were aligned to the reference-sequence using NUCmer alignment generator from MUMmer package [22]. Owing to the chloroplast genome structure (two long inverted repeats) the option to use all matches regardless of the uniqueness was enabled in the script. All other parameters were left as default. Based on the MUMer output the longest contigs with the best mapping results (query coverage, precent identity and alignment length) were chosen as scaffolds and manually joined together by overlapping the remaining short contigs. In cases when there was not enough contigs to reconstruct the chloroplast genome sequence, *de novo* assembly was repeated with different parameter for word size in the CLC-GW and subjected again to the alignment tool. Consensus sequences from contigs mapping and joining were also screened for SNPs and indels based on the reference-sequence.

### Data analysis – variant detection

The consensus sequences generated by both mapping and *de novo* assembly of the reads were imported into Geneious 6.1.6 (www.geneious.com) and each of them was separately mapped to reference-sequence. To generate the alignments the Geneious Map to Reference tool was used with the default parameters. Subsequently the alignments were screened to find variations/SNPs using the tool available in Geneious package.

### Validation of the most probable variant

The wild rice chloroplast consensus sequences derived from reads mapping from Illumina and Ion Torrent platforms were aligned and scanned for SNPs and indels (as described above). If a variant was found in one consensus but was not present in the other it was reported as a discrepancy and closely studied. To confirm which of the disagreeing variants was more likely to be true the following procedure was adopted: (1) visual investigation of mapping results in CLC-GW and (2) aligning the conflicting regions from mapping derived consensus with contigs sequences originated from *de novo* assemblies.

### Data deposition

The chloroplast genome for Australian wild rice genotype sample (W-rice-genotype) has been deposited in GenBank with the sequence accession number KF428978.

## Results

### Mapping and *de novo* assembly of sequence reads

The CLC Genomics Workbench was used to trim sequence reads from both the Illumina and Ion Torrent platforms and then processed, using the mapping and *de novo* assembly mode, to generate a mapping-consensus or *de novo*-consensus chloroplast sequences, respectively. The Torrent Suite Software, a dedicated analysis tool for Ion Torrent reads, was used to trim sequence reads from the Ion Torrent platform and then processed using the reference-guided *de novo* assembly mode (the only available mode) to generate a reference-guided *de novo*-consensus chloroplast sequence. The published chloroplast sequence of the R-rice-genotype (*Oryza sativa* spp. *japonica* var. Nipponbare) was used as a reference chloroplast genome sequence (reference-sequence) for mapping of reads or contigs. Sequencing data of the two rice genotypes, generated by both sequencing platforms, was of good quality with average PHRED scores ranging above 25 (Table S1).

In case of the R-rice-genotype, CLC trimmed-reads from both sequencing platforms mapped to the entire reference-sequence indicating complete reference-sequence coverage. However, the mapping-consensus chloroplast sequences varied in length based on sequencing platform reads. Similar results were obtained for the W-rice-genotype (Table S2).

Using the *de novo* mode in the CLC-GW and reads from both sequencing platforms, a number of contigs were generated with a subset of these mapping to the reference-sequence (Table S3). Similar results were obtained with the Ion Torrent reads when using the reference-assisted *de novo* mode on the Torrent Suite Software (Table S3). For both rice genotypes, contigs, which mapped to the reference-sequence, were checked if contiguous/overlapping to generate a *de novo*-consensus sequence. Using the *de novo* mode in the CLC-GW, respective *de novo*-consensus chloroplast sequences were generated for the reference-rice-genotype and the wild-rice-genotype but only from their respective Ion Torrent reads and not their Illumina reads. Using the reference-assisted *de novo* mode, reference-guided *de novo*-consensus chloroplast sequences were generated for both genotypes.

Thus, all contigs generated from Ion Torrent reads using both assembly algorithms (in the CLC-GW and Torrent Suite Software) could be joined together based on their position and overlapping information creating full length consensus sequences for the chloroplast genome (Table S3). Consensus sequences could not be created from contigs generated in the CLC-GW from Illumina reads for either of the rice genotypes, possibly because of the limitations of read length (Table S3). Although these contigs were well mapped to the reference, there were gaps between some of them that prevented their joining.

### Analysis of reference-rice-genotype

The mapping-consensus chloroplast genome sequence (134,551 bp) generated by mapping the Illumina reads from [11] to the reference-sequence in the CLC-GW was, as expected, identical to the reference-sequence (Table 1). The mapping-consensus sequence generated by mapping of the Ion Torrent reads to the reference-sequence in the CLC-GW was shorter than the reference-sequence by 31 nucleotides with no base mismatches, but diverged at 31 positions comprising of 30 deletions and 1 insertion and where all but one of the polymorphisms were found in homopolymer regions of length from 2 to 9 bases. The *de novo*-consensus sequence built by mapping and joining the contigs from *de novo* assembly of Ion Torrent reads (performed in CLC-GW) was shorter than the reference-sequence by 48 bp with no base mismatches, but diverged at 54 positions comprising of 48 deletions and 6 insertion and where all but two of the polymorphisms (indels) were found in homopolymer regions of length from 2 to 17 identical bases.

**Table 1:** Comparison of chloroplast consensus sequences of the cultivated reference rice genotype (*Oryza sativa* Nipponbare).

Sequencing platform	Source	Sequence length (bp)	Variants	Deletions	Insertions	Mismatches
Used available reference	GenBank (GU592207)	134,551	–	–	–	–
Illumina [11]	mapping- consensus	134,551	0	0	0	0
Ion Torrent	mapping- consensus	134,520	31	30	1	0
	*de novo-* consensus (CLC-GW	134,503	54	48	6	0
	reference-assisted *de novo*- consensus(Torrent Suite)	134,581	50	9	41	0

The reference-guided *de novo*-consensus chloroplast sequence obtained from contigs generated in Torrent Suite Software using Ion Torrent reads was longer than the reference-sequence by 30 bases with no base mismatches, but diverged at 50 positions comprising of 9 deletions and 41 insertions and where all polymorphisms (indels) were found in homopolymer regions of length from 2 to 12 identical bases.

The variants in the mapping-derived consensus sequences generated by the CLC-GW and using the Illumina or Ion Torrent reads were predominantly deletions, which is in contrast to the reference-guided *de novo*-consensus chloroplast sequence generated by the Torrent Suite Software using Ion Torrent reads, where the variants were predominantly insertions. Overall, 135 variants were detected using these different approaches. Among them only 8 were common for all of the methods (the same position, length and type). The rest of the variants were either present in two of the approaches or were unique to only one of them.

**Figure 1:**
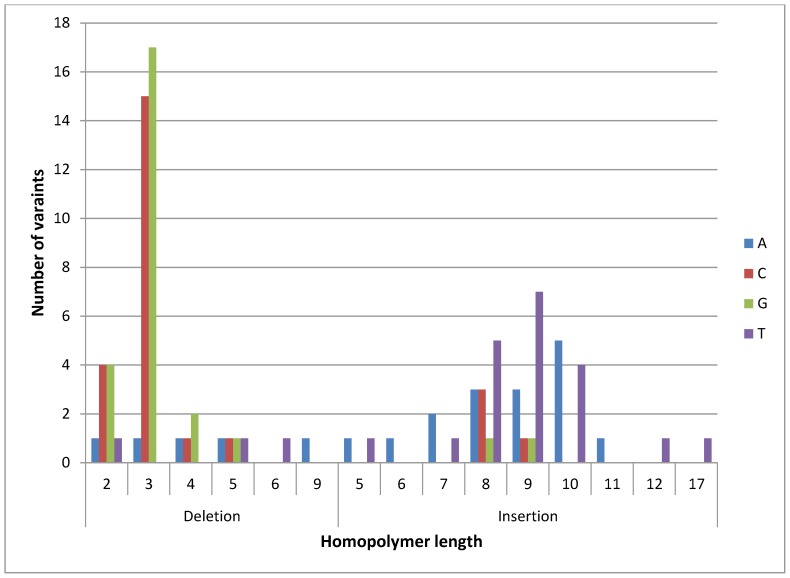
Variants in indels in cultivated (cv. Nipponbare) rice chloroplast consensus. Sequences generated by mapping and assembly of Ion Torrent reads to the available chloroplast sequence in GenBank for this genotype. The number of variants is shown with respect to its type (deletion or insertion) and position (the length of homopolymer region where the variants were found).

The analysis of regions in the chloroplast genome where the indels were found in the Ion Torrent data (both mapping and assembly consensus) revealed biases in terms of polymorphism nature, nucleotide type and location. Data from all approaches were merged and duplicated regions were removed. The number of indels that involved C/G bases was slightly higher than A/T bases (53 and 44, respectively). It is worth noting that the GC content of the chloroplast genome is 39%. Also, more deletions than insertions were observed (except for the consensus from the Torrent Suite Software assembly) and the majority were of G/C nucleotides in short homopolymeric regions (2–5 bp long with the vast majority of these variants occurring in 3 bp long homopolymer) (Figure 1). On the other hand, insertions were found mostly in longer homopolymers (5–17 bp long) and involved almost only A/T nucleotides.

### Analysis of wild-rice-genotype

The reference-sequence was used to generate consensus chloroplast sequences using mapping or *de novo* approaches. For variant analysis, consensus sequences were compared to the reference-sequence unless indicated otherwise. The mapping-consensus sequence generated by mapping the Illumina reads of the wild rice plant using the CLC-GW was shorter than the reference-sequence by 20 bases (Table 2). In comparison, there were 128 variants found between the two sequences, which included 18 deletions, 13 insertions and 97 mismatches. Among mismatches 92 were single-nucleotide variants (SNPs) (48 transitions and 44 transversions) and 5 were multi-nucleotide variants (MNVs). The mapping-consensus obtained by mapping a subset of Illumina reads differed from the previous sequence by one deletion of 2 bases (Table 2), although, this variation was not found in the reference-sequence.

**Table 2:** Comparison of chloroplast consensus sequences of the wild rice (*Oryza rufipogon*-like).

Sequencing platform	Source	Sequencelength (bp)	Variants	Deletions	Insertions	Mismatches
Used available reference	GenBank (GU592207)	134,551	–	–	–	–
Illumina	mapping- consensus	134,531	128	18	13	97
	mapping-consensus (subset of reads)	134,529	129	19	13	97
Ion Torrent	mapping consensus	134,525	139	30	14	95
	*de novo-* consensus (CLC-GW)	134,521	155	43	17	95
	reference-assisted *de novo*- consensus(Torrent Suite)	134,554	147	23	28	96

The consensus sequence generated by mapping Ion Torrent reads of the wild-rice-genotype using the CLC-GW was shorter than the reference-sequence by 26 bases and shorter by 6 bases than the consensus sequence from Illumina sequencing. The two sequences differed by 139 variants, which included 30 deletions, 14 insertions and 95 mismatches. Among the mismatches 90 were SNPs (47 transitions and 43 transversions) and 5 were MNVs.

As was observed for the R-rice-genotype, the de novo-consensus sequence differed from the reference-sequence at more positions than the mapping-consensus sequence (Table 1 and Table 2). In addition, a higher number of deletions were observed in consensus sequences obtained from the CLC-GW analysis as compared to the Torrent Suite Software analysis where more insertions were observed (Table 1 and Table 2).

### Comparison of sequencing platforms

The mapping-consensus chloroplast sequences of the wild-rice-genotype generated in the CLC-GW using the Illumina and Ion Torrent reads were used to compare sequence platforms. When comparing variations found in the wild-rice-genotype by both sequencing technologies, 20 discrepancies (variant in one consensus not present in the other) were observed (Table 3). However, in the Ion Torrent consensus sequence there were fifteen variants and in the Illumina consensus there were only three. Moreover, two variants found at the same position differed in nucleotide composition in both consensus sequences.

**Table 3:** Inconsistent variations found in wild rice chloroplast mapping-consensus sequences and their validation.

Variations		Reference-sequence		Mapping-consensus sequence		The most probable variant
No	Type	Position	Allele	HiSeq	Ion Torrent	
#1	MNV	57,036	TT	TT	AA	TT
#2	ins	65,465∧65,466	−	−	TCCTATTTAATA	TTCCTATTTAATA
#3	MNV	66,897	CGAT	TAGA	CGAT	TAGAAATAAAAAATTCTAA
#4	SNP	66,902	C	A	C	TAGAAATAAAAAATTCTAA
#5	SNP	17,366	T	A	T	T
#6	SNP/del	17,368	C	A	−	−
#7	ins	3,545∧3,546	−	AA	A	−
#8	del	21,808	C	C	−	C
#9	del	57,027	T	T	−	T
#10	del	81,342	G	G	−	G
#11	del	91,427	C	C	−	C
#12	del	91,589	C	C	−	C
#13	del	97,135	G	G	−	G
#14	del	111,639	G	G	−	G
#15	del	116,139	C	C	−	C
#16	del	118,025	C	C	−	C
#17	del	119,245	G	G	−	G
#18	del	122,914	C	C	−	C
#19	del	123,568	G	G	−	G
#20	del	133,816	C	C	−	C

Variations derived by Illumina and Ion Torrent sequencing.

SNP – single-nucleotide variant, MNV – multi-nucleotide variant, ins – insertion, del – deletion.

**Figure 2:**
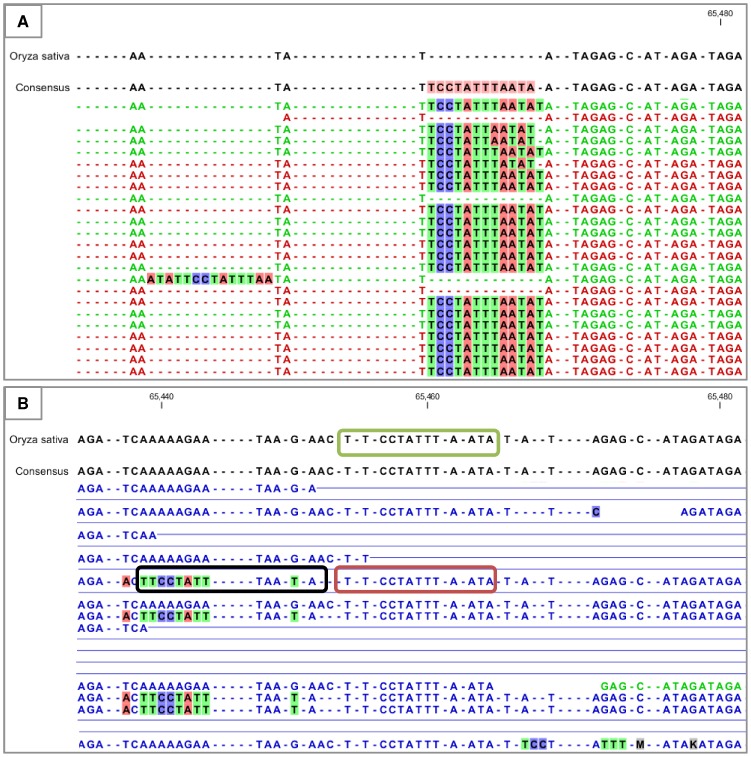
Snapshot of mapping results of wild rice Ion Torrent (A) and Illumina (B) reads. Reads were mapped to the chloroplast reference of *Oryza sativa* cv. Nipponbare. In the mapping of Ion Torrent reads there was a long insertion (TCCTATTTAATA) reported in the consensus sequence of wild rice chloroplast ((A), marked with orange background colour). This insertion was missed in the mapping of Illumina reads, although it was present in the reads ((B), example of the read sequence marked in black rectangle). The nucleotides in the insertion were duplicated in wild rice (sequence marked in red rectangle), and not in the reference genome where only one copy of these nucleotides was present (marked in green rectangle). The duplicated region was a probable cause of the misalignment of reads. Oryza sativa – fragment of chloroplast sequence of *Oryza sativa* spp. *japonica* var. Nipponbare; Consensus – consensus sequence of wild rice chloroplast sequence derived by mapping reads from Illumina (A) and Ion Torrent (B) platforms to the reference. Nucleotides with background colours represent the mismatches between reads and the reference sequence; paired end reads are shown in blue; single reads are shown in green and red (in forward and reverse orientation, respectively).

**Figure 3:**
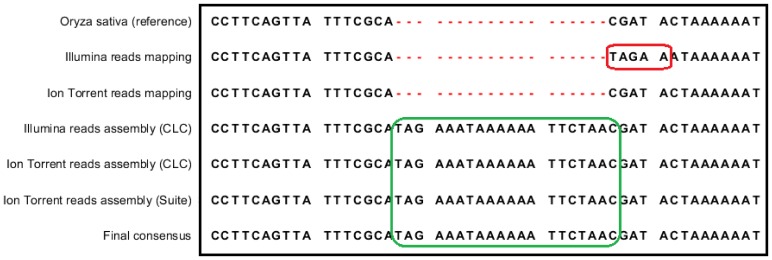
Alignment of regions #3 and #4 from Table 3 showing discrepancies in consensus sequences. The fragment circled in red shows false called SNPs (#3 and #4, Table 3, Illumina consensus); these SNPs were incorrect because of the long insertion present in wild rice sequence but not in the reference. The fragments circled in green illustrate this long insertion found in wild rice chloroplast genome by means of reads assembly from both platforms and both assembly tools. Final sequence was created based on this information. Oryza sativa (reference) – region 66860.66940 from chloroplast sequence of *Oryza sativa* spp. *japonica* var. Nipponbare; Illumina reads mapping and Ion Torrent reads mapping – regions from consensus sequence generated by mapping wild rice Illumina and Ion Torrent reads, respectively, to the reference sequence; Illumina reads assembly and Ion Torrent reads assembly – regions from contigs generated by assembly of reads from Illumina and Ion Torrent platforms, respectively; CLC – assembly performed in CLC Genomic Workbench; Suite – assembly performed in Torrent Suite Software; Final consensus – final wild rice chloroplast genome sequence (GenBank accession – KF428978).

Read mapping files derived from both mapping-consensus sequences were visually inspected to check for reads mapped to all 20 positions to determine sequence read error or for mapping error. The region in the Ion Torrent mapping-consensus sequence with the substitution of TT → AA (Table 3, variant #1) had several mapped reads with many mismatches. Reads from this region when extracted and blasted against the nucleotide sequence collection (nr/nt) at NCBI website (http://blast.ncbi.nlm.nih.gov) gave hits to the chloroplast but also to the nuclear DNA of *Oryza* species. In addition, this variant was not observed in the corresponding region of the contig sequences obtained from *de novo* assembly (from both analysis software), indicating that the variant was not due to sequencing error but due to mapping artefact. Similarly, mapping error and not sequence read error was the reason for variants detected at several positions in one or the other mapping-consensus sequences (Table 3: for # 2 see Figure 2, for #3 and #4 see Figure 3), as these variants were not observed in the corresponding region of the contig sequences obtained from *de novo* assembly (from both analysis software). One of the variants (#7), an insertion of an A in the Ion Torrent consensus and AA in the Illumina consensus, was found in a long homopolymer stretch of 10 A’s. The location of the variation suggested that in both cases it could be an error. Variations at this position in contigs sequences varied from 2 to 3 A’s insertions. This polymorphism was not called in the consensus from the subset of Illumina data. Comparison of other chloroplast genomes, known to have been sequenced on the Illumina platform (GAII) [11], showed some to have this insertion, namely Australian *Oryza rufipogon* (GenBank accession – JN005833), Asian *Oryza rufipogon* (GenBank accession – JN005832) and *Oryza meridionalis* (GenBank accession – JN005831). Interestingly, the insertion was not present in the chloroplast genome sequence of *Oryza sativa* spp. *japonica* var. Nipponbare (reference-sequence), *Oryza sativa* ssp. *indica* isolate 93-11 (GenBank accession – AY522329) and *Oryza nivara* (GenBank accession – AP006728) which were sequenced by Sanger technology. Thus for this variant we cannot conclude with certainty if this discrepancy is due to mapping artefact or due to read error. However, all other errors found in Ion Torrent sequence (Table 3, #8 to #20) were not due mapping errors but read errors from either deletions or insertions in homopolymer regions.

Based on the analysis and all the findings using various approaches outlined above, an amended wild-rice-genotype chloroplast consensus sequence was created incorporating the most probable variants from Table 3. To assess the correctness of this amended chloroplast sequence paired-end reads from Illumina sequencing platform were re-mapped to this new wild rice chloroplast genome. Illumina reads as against Ion Torrent reads of the W-rice-genotype were selected for remapping as even short Illumina reads (36 bases) of the R-rice-genotype provided a consensus matching the reference-sequence (Table 1) [11], as against an inaccurate consensus when Ion Torrent reads were used (Table 1) due to indel-associated errors. The new mapping results for the wild rice show only one discrepancy described earlier (Table 3, #7) in a long homopolymer region. All other sites were identical giving an additional assurance of the correctness of the final consensus.

## Discussion

Various NGS technologies are now available for the rapid sequencing of whole genomes [17], [23], [24]. The choice of selecting one or more NGS technologies depends on the yield of data required, read length required, cost per data point and accuracy of the sequence data [25]. Systematic as against random errors can compromise the use of sequence data [26], [27]. In this study we analysed NGS reads of two rice genotypes obtained from the Ion Torrent platform and compared it for accuracy to those obtained from the Illumina platform. A comparison of the three sequencing systems is presented in Table 4.

**Table 4:** The comparison between the tree sequencing systems utilised in the study.

	Illumina GAIIx	Illumina HiSeq2000	Ion Torrent PGM −318 chip
Sequencing method	Synthesis (light detection)	Synthesis (light detection)	Synthesis (proton detection)
Amplification method	Bridge PCR	Bridge PCR	Emulsion PCR
Read length^a^	Up to 2×150 bp	1×50 bp, 2×50 bp, 2×100 bp	∼200 bp, ∼400 bp
Paired reads	Yes	yes	Yes
Insert size	Up to 700 bp	Up to 700 bp	Up to 250 bp
Output data/run	30 Gb	600 Gb	Up to 2 Gb
Time/run^b^	10–14 days	8–11 days	4–7 hours
Cost/Gb^c^	$148	$41	$1000
Instrument cost	$256 K	$654 K	$80 K
Accuracy	>99.9%	>99.9%	99%
Error rate^d^	≥0.1%	≥0.1%	∼1%
Primary errors	Substitutions	Substitutions	Indels
DNA requirements	0.05–1 ug	0.05–1 ug	0.1–1 ug

Gb – gigabase, bp – base pair, K – thousand, uq - microgram.

a Annotation ‘2 x’ refers to paired end reads and ‘1 x’ to single reads.

b Run time from minimum to maximum read lengths.

c Includes one sample and one sequencing kit per run.

d percentage of errors per base in single read.

The Ion Personal Genome Machine (PGM) is characterised by long read length, very short run times and inexpensive consumables [28]. The drawbacks include low output, high cost per Gb (gigabase) of sequence [25] and the longer time required for the library preparation relative to that required for the sequencing run. Moreover, the PGM produces biased sequences from high homopolymer regions, which result in indel errors [27]. However, this is a developing platform with high potential for improvement in accuracy, read length and cost per read and per Gb. As the machine is the chip, it can be easily upgraded by the release of new chips. The PGM was the first next-generation sequencer, which price that dropped below $100 K. At the time of the study the PGM cost was around $80 K, which included the instrument, temple preparation system, enrichment system and server. The main advantages of HiSeq2000 system are the high throughput, large output data volumes per run and low cost of reagents per Gb [29]. The shortcomings of the platform embrace short read length and the need for advanced computational resources to process and store the enormous data volumes created from each run. Furthermore, the run time is considerably longer than that for the PGM, and the Illumina has a much higher initial capital cost. The specification of the GAIIx system was very similar to the HiSeq2000 with smaller output per run, higher cost per Gb and significantly lower instrument cost. The sequencing accuracy on both Illumina platforms is high with the most commonly encountered errors being substitutions. A wide range of Illumina instruments are now available with differing data volumes, running times and costs.

We selected the cultivated rice genotype (cv. Nipponbare) as one of the samples as its chloroplast sequence, derived by Sanger sequencing and considered as accurate, was available and could be used as reference sequence to check for errors in consensus sequences derived from NGS sequencing on both platforms. The aim was to generate both mapping and *de novo*-consensus sequences to determine read accuracy of both platforms.

We were unable to obtain *de novo*-consensus sequences for both genotypes with Illumina reads when processed by CLC-GW as the contigs generated were non-contiguous when mapped to the reference-sequence. In the case of reference-genotype, read coverage depth and read length contributed to non-contiguous contigs as the read depth in regions corresponding to gaps was between 7 and 30, when examined for base calling and read mapping depth to the mapping-consensus, which is well below the average mapping read coverage depth of 127.7 (Table S2). In the case of wild-rice-genotype with longer read length (100 bases), read length but not read coverage depth may have contributed to non-contiguous contigs as the read depth in regions corresponding to the gaps was between 7,000 to 11,000, when examined for base calling and read mapping depth to the in the mapping-consensus, which is well above the average mapping read coverage depth of 8,143.2 (Table S2). Interestingly, *de novo*-consensus, although not accurate (Table 1) was generated for the reference-genotype using CLC-GW and the longer (200 bases) Ion Torrent reads. We can thus conclude that read lengths of more than 100 bases would be required to successfully obtain a *de novo*-consensus of the chloroplast genome.

In case of the reference-genotype, mapping-consensus sequences were generated using reads from both platforms, with no mismatches. However, mapping-consensus sequences showed several indels due to inaccuracies in the Ion Torrent reads mainly in homopolymer regions. In case of the short Illumina reads (36 bases) of the R-rice-genotype, neither the read length (36 bases) nor the sequence yield (minimum reference-sequence read mapping coverage of 7) was a limitation in generating an accurate mapping-consensus when processed by the CLC-GW, indicating the accuracy of the Illumina sequencing platform. In case of the longer Ion Torrent reads (200 bases), when processed by the CLC-GW and the Torrent Suite Software, consensus-sequences with no mismatches were generated but with several variants all of which were indels and predominantly in homopolymer regions (Table 1). These results indicate the actual presence of these anomalous indels in the Ion Torrent reads which contributed to the inaccuracy in the consensus sequence generated and to the unreliability of any indels calls made when using these reads. It is known that the Ion Torrent sequencing platform generates anomalous indels [27]. Interestingly, the preference of the CLC-GW and the Torrent Suite analysis tool in filtering out predominantly either insertions or deletion, respectively, as observed in both rice genotypes (Tables 1 and 2), indicates the possibility of these analysis tools to be amended to deal with anomalous indels in the Ion Torrent reads. We also observed a bias for indels in homopolymer regions, where deletions occurred in shorter regions while insertions occurred in longer regions. These findings are inconsistent with a recent study on Ion Torrent sequencing bias where it was reported that an increased deletion rate was positively correlated with increased lengths of homopolymer regions and that insertions were independent of homopolymer length [27], [30]. This is probably because of the different genomes used or that the chloroplast genome is AT-rich and the longest C/G stretches found are 9 bp long (2 regions), 8 bp (4 regions) and 7 bp (9 regions).

The systematic pattern of bias, specific types of nucleotide variants in long versus short repeats, in the single base indels in homopolymers (Figure 1) is likely to be a feature of chloroplasts with their specific patterns of homopolymer repeat frequency. However, systematic errors reported in the Ion Torrent data in earlier studies [27] were not found in this analysis of the chloroplast possibly because we used a more recent version of the technology. We can conclude that both sequencing platforms produce high quality data with relatively low rates of discrepancy in the calling of polymorphisms, especially SNPs, but the Ion Torrent data would be less reliable for indel calls.

We compared the two sequencing platforms in generating an accurate consensus chloroplast sequence by comparing the mapping-consensus sequences generated for both genotypes. For the R-rice genotype, no mismatches were observed indicating the two platforms to be comparable for SNP calls as has been reported for microbial genomes [25]. When comparing the mapping-consensus sequences of the W-rice-genotype we identified twenty discrepancies. It must be noted that some of the discrepancies were caused by misalignment of reads and as has been previously recognized [31] there is an important issue with mismatches in close proximity of indel events. Some read mapping tools can have problems with these regions resulting in false SNPs calling. However, for both the rice genotypes we observed erroneous indel calls with Ion Torrent reads mainly in homopolymer regions.

The importance of obtaining a *de novo* sequence was clear from our study as discrepancies observed in the mapping sequences of the W-rice-genotype were curated using the *de novo* contigs. The *de novo* assembly of chloroplast genomes using the tools applied in this study was easier with the longer Ion Torrent data than with the Illumina data. Although increasing number of reference genome sequences are being generated to support chloroplast genome analysis, the ability to generate whole chloroplast genome sequences *de novo* will find wide application. The first report of total plant DNA analysis for chloroplast sequencing [11] used short Illumina reads (36 bp) and relied on a reference genome for successful assembly. More recent studies based upon longer reads [12], [23] have reported more success with *de novo* assembly.

## Conclusions

This study demonstrates that *de novo* assembly of an accurate whole chloroplast genome sequence will be possible for routine plant barcoding. Analysis of plants based upon appropriate and careful analysis of shotgun sequencing of total DNA promises to provide a barcode that will have wide application. Having a well curated and reliable consensus chloroplast sequence for a plant sample provides greater certainty of obtaining reliable results in many critical studies such as plant identification, purity assessment, phylogenetic analysis and heteroplasmy analysis. Continuing improvements in sequencing platforms and analysis tools will make this method more reliable and cost effective for a wide range of research and industrial applications. The significant advances in these competing sequencing platforms that have been foreshadowed by the manufactures promise dramatic reductions in cost in the near future. This would make barcoding by sequencing the whole chloroplast in this way the preferred option for plant identification in many research and industrial applications. A comparison of the platforms at the same level of coverage is useful in providing a guide to likely comparative performance as the number of reads on these platforms increases in the future. Costs will change for each platform as read length and read volume are increased.

However cost is not the only advantage of this approach. The use of total DNA from the sample without amplification simplifies the analysis but also provides greater opportunity to measure in an unbiased way the contribution of different genotypes in complex mixtures. The risks of preferential amplification or enrichment of chloroplasts from a specific genotype that complicated earlier analysis will be avoided.

## Supporting Information

Table S1 **Summary statistics of raw reads obtained from PGM Ion Torrent and Illumina platforms for rice (** ***Oryza sativa*** **spp.** ***japonica*** **var. Nipponbare) and wild rice (** ***Oryza rufipogon*** **-like plant from Australia).** Quality distribution is represented as PHRED scores.
(PDF)

Table S2 **Mapping statistics of CLC trimmed reads from Illumina and PGM Ion Torrent platforms.**
(PDF)

Table S3 **Assembly results performed on CLC Genomic Workbench and Torrent Suite Software of reads from Illumina and Ion Torrent platforms and mapping results of generated contigs to chloroplast reference-sequence.**
(PDF)
