# Supplementary material for: Direct Chloroplast Sequencing: Comparison of Sequencing Platforms and Analysis Tools for Whole Chloroplast Barcoding
Source: PLoS One. 2014 Oct 17;9(10):e110387. doi: 10.1371/journal.pone.0110387 (PMC4201551; doi:10.1371/journal.pone.0110387)
Supplement: Table S1 — Summary statistics of raw reads obtained from PGM Ion Torrent and Illumina platforms for rice ( Oryza sativa spp. japonica var. Nipponbare) and wild rice ( Oryza rufipogon -like plant from Australia). Quality distribution is represented as PHRED scores. (PDF) [file pone.0110387.s001.pdf]

Supplementary Table S1. Summary statistics of raw reads obtained from PGM Ion Torrent and Illumina platforms for rice (*Oryza sativa* spp. *japonica* var. Nipponbare) and wild rice (*Oryza rufipogon*-like plant from Australia). Quality distribution is represented as PHRED scores.

	**Num. of reads**	**Approach**	**Length distribution**	**Length avg.**	**GC avg. [%]**	**Quality distribution**	**Quality avg.**
Oryza sativa spp. *japonica* var. Nipponbare							
PGM Ion Torrent	6,655,114	Single reads	8-376	204	41.2	8-35	26
GAII Illumina [11]	9,689,084 (4,844,542 each pair)	Paired-end reads	36	36	46.3	2-34	29
Wild rice *(Oryza rufipogon*-like*)*							
PGM Ion Torrent	5,732,260	Single reads	8-362	189	41.3	6-36	25
HiSeq Illumina	390,365,066 (195,182,533 each pair)	Paired-end reads	100	100	41.5	2-40	32
