# Supplementary material for: Direct Chloroplast Sequencing: Comparison of Sequencing Platforms and Analysis Tools for Whole Chloroplast Barcoding
Source: PLoS One. 2014 Oct 17;9(10):e110387. doi: 10.1371/journal.pone.0110387 (PMC4201551; doi:10.1371/journal.pone.0110387)
Supplement: Table S2 — Mapping statistics of CLC trimmed reads from Illumina and PGM Ion Torrent platforms. (PDF) [file pone.0110387.s002.pdf]

Supplementary Table S2. Mapping statistics of CLC trimmed reads from Illumina and PGM Ion Torrent platforms.

	***Oryza sativa* spp. *japonica* var. Nipponbare**		**Wild rice (*Oryza rufipogon*-like)**		
	**GAII Illumina**	**PGM Ion Torrent**	**HiSeq Illumina**	**HiSeq Illumina subset**	**PGM Ion Torrent**
Mapped reads (Forward/Reversed)	525,452 (262,567/262,885)	223,605 (112,154/111,451)	12,424,965 (6,211,497/6,213,468)	327,403 (164,024/163,898)	169,665 (85,317/84,348)
% mapped reads	5.42	3.39	3.43	3.46	3.17
Non-specific matches (% non-specific matches)	204,877 (39.9)	64,560 (28.9)	3,601,138 (29.0)	93,502 (28.5)	50,521 (29.8)
Non-perfect matches (% non-perfect matches)	18,385 (3.5)	164,176 (73.4)	1,745,820 (14.1)	47,444 (14.5)	130,855 (77.1)
Min coverage	7	96	1,203	75	69
Max coverage	688	534	13,231	360	431
Average coverage	127.7	333.4	8,143.2	218.4	243.6
% GC	39	39	39	39	39
Consensus length	134,551	134,520	134,531	134,529	134,525
