# Supplementary material for: Direct Chloroplast Sequencing: Comparison of Sequencing Platforms and Analysis Tools for Whole Chloroplast Barcoding
Source: PLoS One. 2014 Oct 17;9(10):e110387. doi: 10.1371/journal.pone.0110387 (PMC4201551; doi:10.1371/journal.pone.0110387)
Supplement: Table S3 — Assembly results performed on CLC Genomic Workbench and Torrent Suite Software of reads from Illumina and Ion Torrent platforms and mapping results of generated contigs to chloroplast reference-sequence. (PDF) [file pone.0110387.s003.pdf]

Supplementary Table S3. Assembly results performed on CLC Genomic Workbench and Torrent Suite Software of reads from Illumina and Ion Torrent platforms and mapping results of generated contigs to chloroplast reference-sequence.

Sequencing platform	Assembly method	Assembly tool	Overall number of contigs	Contigs mapped to chloroplast genome$^{\pm}$	Contigs chosen to create consensus sequence
Oryza sativa spp. *japonica* var. Nipponbare					
GAII Illumina	*de novo*	CLC-GW	961	25	NA
PGM Ion Torrent	*de novo*	CLC-GW	268,869	116	25
PGM Ion Torrent	reference-assisted	Torrent Suite	213*	34	2
Wild rice *(Oryza rufipogon*-like*)*					
HiSeq Illumina	*de novo*	CLC-GW	224,756	94	NA
PGM Ion Torrent	*de novo*	CLC-GW	227,610	99	19
PGM Ion Torrent	reference-assisted	Torrent Suite	222*	31	2

NA – not applicable, corresponds to method that could not be used to create consensus sequence because of the gaps between contigs and reference genome, detailed description in the text;

* - in Torrent Suite Software the assembly was run in the reference-assisted mode using chloroplast reference and the number of contigs given corresponds to contigs assembled based on the chloroplast sequence not the entire genome;

$^{\pm}$ - numbers correspond to all contigs mapped to the reference genome by means of MUMmer with default parameters and all matches regardless of their uniqueness, duplicated contigs are not reported.
